# Anti-Obesity Medications in Longevity and Aesthetic Medicine

**DOI:** 10.3390/jcm15156026

**Published:** 2026-08-03

**Authors:** Julia Bijoch

**Affiliations:** Faculty of Medicine, Collegium Medicum, WSB University, 41-300 Dabrowa Gornicza, Poland; julia.bijoch@wsb.edu.pl

**Keywords:** anti-obesity medications, GLP-1 receptor agonists, tirzepatide, semaglutide, longevity, biological age, body composition, aesthetic medicine

## Abstract

Anti-obesity medications (AOMs), led by the glucagon-like peptide-1 receptor agonists (GLP-1 RAs) semaglutide and liraglutide and the dual GIP/GLP-1 receptor agonist tirzepatide, have produced substantial weight reduction and growing signals of benefit that extend well beyond adiposity. These agents are now discussed in relation to longevity and aesthetic medicine, raising the question of whether their effects reach ageing biology and appearance. This narrative review (PubMed, EMBASE, Scopus, Web of Science, Cochrane Library, and Clinical trial registries; January 2010–June 2026) integrates mechanistic studies, randomised cardiovascular and renal outcome trials, ageing-biomarker analyses, body-composition data, and patient-facing aesthetic phenomena. The evidence indicates that AOMs reduce major cardiovascular events, slow kidney and liver disease progression, and lower all-cause mortality in selected populations; exploratory proteomic and epigenetic analyses further suggest effects partly independent of weight loss, though these do not establish slowed ageing. Newer multi-receptor agents act with greater metabolic specificity: glucagon-containing agents such as survodutide and the triple agonist retatrutide preferentially reduce visceral and hepatic fat (liver-fat reductions of roughly 60–80% in places), a quality of weight loss arguably more relevant to healthspan than its quantity. Concurrently, rapid large-magnitude weight loss drives soft-tissue and appearance changes colloquially termed “Ozempic face” and “Ozempic body”, alongside accelerated skin laxity and loss of lean mass, the latter tempered by data showing lean-loss proportions comparable to established agents.

## 1. Introduction

The global obesity epidemic, affecting more than one billion people, has driven an era of rapid pharmacological innovation [1]. Anti-obesity medications (AOMs) that exploit the biology of glucagon-like peptide-1 (GLP-1) and glucose-dependent insulinotropic polypeptide (GIP) have become the leading pharmacologic strategy for obesity and type 2 diabetes mellitus (T2DM). It should be noted, however, that the term anti-obesity medication is broader than the incretin-based therapies: several other agents with distinct mechanisms of action remain approved by the U.S. Food and Drug Administration (FDA) and the European Medicines Agency (EMA) for the treatment of obesity, including orlistat, the combinations naltrexone/bupropion and phentermine/topiramate, and, historically, other centrally acting agents. Their clinical use has nonetheless declined substantially in recent years, owing to their more modest efficacy and less favourable tolerability relative to the GLP-1 receptor agonist-based agents that are the focus of this review. In the pivotal STEP 1 trial, once-weekly semaglutide 2.4 mg produced a mean weight reduction of 14.9% at 68 weeks [2], and in SURMOUNT-1 the dual GIP/GLP-1 receptor agonist tirzepatide produced reductions of up to 22.5% [3]. The SELECT trial subsequently demonstrated a 20% relative reduction in major adverse cardiovascular events with semaglutide 2.4 mg in patients with obesity but without diabetes [4], while dual and triple agonists have shown efficacy in obesity-related complications, including obstructive sleep apnoea with tirzepatide in SURMOUNT-OSA [5] and a range of weight-related endpoints with retatrutide in the phase 3 TRIUMPH programme [6], extending the rationale for these agents well beyond weight management. Recent international guidance reflects this broader, more individualised approach: the 2026 update of the European Association for the Study of Obesity (EASO) framework recognises obesity as a heterogeneous disease whose complications extend beyond classical cardiometabolic risk factors, and structures pharmacological treatment around the presence or absence of specific obesity-related conditions rather than weight alone [7].

Alongside this clinical evidence, a broader idea has entered both scientific and public discourse: that the benefits of these agents may extend beyond weight and metabolic disease to influence ageing itself, and that they may reshape appearance in ways relevant to aesthetic medicine. This notion—sometimes expressed in the suggestion that weight loss could eventually become secondary to a wider role in preventing or delaying age-related disease—has been voiced in commentary surrounding major scientific meetings and in the press, and it usefully anchors the questions this review addresses. It is, however, only a starting point. The remainder of this review sets aside that framing and independently examines what the scientific evidence does and does not support regarding the longevity and aesthetic dimensions of AOMs, distinguishing throughout between mechanistic plausibility, preclinical findings, and demonstrated clinical effect.

In parallel, the same agents have become central to aesthetic medicine. The scale and visibility of the AOM-treated population have made rapid, large-magnitude weight loss—and its visible consequences for the face, skin, and body—a mainstream concern, generating patient-facing terms such as “Ozempic face” and “Ozempic body” and driving demand for volume restoration, skin tightening, and body contouring [8,9]. The longevity and aesthetic dimensions are therefore intertwined: both follow from the same pronounced shifts in metabolism and body composition that these drugs produce.

This review summarises the evidence on how established AOMs (semaglutide, tirzepatide, liraglutide, and dulaglutide) and the emerging multi-receptor pipeline (triple agonists, GLP-1/glucagon co-agonists, amylin-based combinations, and GIP-antagonist hybrids) intersect with longevity and aesthetic medicine, and provides considerations for clinicians [10]. Throughout, a careful distinction is drawn between mechanistically plausible hypotheses, preclinical findings, and demonstrated clinical effects because the evidence base, while growing rapidly, remains immature—and the commercial and cultural enthusiasm around “longevity” substantially outpaces it.

## 2. Methods

This is a narrative review. PubMed, EMBASE, Scopus, Web of Science, the Cochrane Library, and ClinicalTrials.gov were searched for records published between January 2010 and June 2026. Search terms combined drug-class descriptors (“GLP-1 receptor agonist”, “incretin”, “anti-obesity medication”, “semaglutide”, “tirzepatide”, “retatrutide”, “cagrilintide”, and “amylin”) with longevity and aesthetic descriptors (“longevity”, “healthspan”, “biological age”, “ageing clock”, “senescence”, “all-cause mortality”, “sarcopenia”, “skin laxity”, “facial volume”, and “aesthetic”). Reference lists of retrieved articles, relevant society guidance, conference proceedings from major diabetes and obesity meetings, and—given the prominence of patient-facing terminology—representative lay and clinical commentary on “Ozempic face” and related phenomena were additionally screened; the latter are treated as hypothesis-generating rather than as evidence. Because dedicated longevity-endpoint trials of AOMs are largely absent, much of the human evidence presented is indirect (derived from cardiovascular, renal, and metabolic trials) or preclinical. The strength of evidence for each clinical consideration is labelled using a simplified Grading of Recommendations, Assessment, Development and Evaluation (GRADE)-informed scheme (see Section 12).

## 3. AOM Pharmacology and the Multi-Receptor Landscape

### 3.1. GLP-1 and GIP Physiology

GLP-1 is a proglucagon-derived peptide secreted from intestinal L-cells; its principal actions are glucose-dependent insulin secretion, glucagon suppression, delayed gastric emptying, and central appetite suppression. Native GLP-1 is degraded within minutes by dipeptidyl peptidase-4 (DPP-4), so therapeutic agents are structurally modified for resistance to degradation and prolonged half-life [11]. GIP, secreted from duodenal K-cells, also potentiates insulin secretion; co-targeting GIP and GLP-1 receptors (as in tirzepatide) yields metabolic effects exceeding selective GLP-1 agonism [12,13]. The GLP-1 receptor is a class B G-protein coupled receptor signalling chiefly through cAMP–protein kinase A (PKA) pathways, and its expression in extra-pancreatic tissues—vasculature, myocardium, kidney, liver, adipose tissue, skin, and central nervous system—underlies the broad physiological reach of these drugs and the plausibility of effects beyond weight [11].

### 3.2. Agents with Evidence Bearing on Longevity and Appearance

The agents discussed in this review are restricted to those for which evidence—clinical or preclinical—bears specifically on longevity, organ protection, or body composition and appearance. Many compounds in development have demonstrated efficacy limited to weight reduction; their inclusion in a longevity- and aesthetics-focused analysis would imply an evidence base that does not presently exist. Accordingly, Table 1 lists the relevant agents by the type and strength of the supporting evidence rather than by receptor mechanism alone.

The strength of supporting evidence differs markedly across these agents. Randomised outcome data bearing on ageing-related morbidity and mortality are available for semaglutide and, following the 2025 publication of SURPASS-CVOT, tirzepatide [14]; liraglutide is supported by earlier cardiovascular-outcome data together with preclinical mechanistic work. For the remaining agents, the longevity- and aesthetics-relevant evidence is either preclinical (the lean-mass preservation reported for amylin analogues [15]) or extrapolated from organ-specific endpoints such as hepatic fat (the glucagon-containing agents). Across all agents, effects on biological ageing itself—as distinct from disease risk or body composition—remain unproven, and the agents producing the greatest weight loss also carry the greatest risk of the lean-mass and soft-tissue changes that may counteract healthy ageing and appearance.

### 3.3. GLP-1 Receptor Expression in Tissues Relevant to Ageing and Appearance

GLP-1 receptor expression has been demonstrated in the myocardium and vasculature, the kidney, the liver, adipose tissue, skin and dermal fibroblasts, and multiple central nervous system regions, providing a mechanistic basis for organ-level effects beyond glucose and weight [11,16]. Receptor activation has been linked in preclinical models to reduced oxidative stress, attenuated low-grade inflammation (“inflammaging”), improved endothelial function, and modulation of cellular senescence and autophagy pathways—hallmarks of biological ageing [16,17]. Whether these receptor-mediated effects are present and functionally significant in the corresponding human tissues at therapeutically relevant drug concentrations, and whether they are separable from the effects of weight loss itself, has not been definitively established and should be regarded as an open question rather than settled fact.

## 4. Cardiovascular, Renal, and Metabolic Organ Protection

The strongest evidence that AOMs do more than reduce weight comes from large randomised outcome trials. In SELECT, semaglutide 2.4 mg reduced major adverse cardiovascular events by 20% in patients with obesity and established cardiovascular disease but without diabetes [4]. The dual agonist tirzepatide was subsequently shown in SURPASS-CVOT to be non-inferior to dulaglutide for major cardiovascular events while reducing all-cause mortality and better preserving renal function over four years, consolidating an organ-protective profile for the class [14]. In the kidney, the FLOW trial of semaglutide in chronic kidney disease and T2DM was stopped early for efficacy, reporting a significant reduction in kidney-disease progression and cardiovascular and renal death [18]. In the liver, GLP-1 RAs and glucagon co-agonists improve metabolic dysfunction-associated steatotic liver disease (MASLD) and steatohepatitis, organs whose function declines with age [19,20]. Crucially, several of these benefits appear, at least in part, before or out of proportion to weight loss, suggesting direct receptor-mediated tissue effects rather than purely the downstream consequences of reduced adiposity [4,21].

From a geroscience standpoint, this matters because cardiovascular, renal, hepatic, and metabolic decline are among the principal drivers of age-related morbidity and mortality. An agent that slows progression across several of these systems simultaneously fits the profile of a candidate “geroprotector”—a drug that targets ageing biology rather than a single disease. However, demonstrating organ protection in high-risk populations is not the same as demonstrating that the underlying ageing process is slowed, and the distinction is central to the cautious interpretation that follows.

## 5. Biological Age, Ageing Clocks, and Mortality

The most provocative recent claims concern biological age. To interpret them, it is useful first to define the tools involved. Ageing clocks are biomarker-based estimators, derived from proteomic, epigenetic (DNA-methylation), or composite panels, that compare a person’s chronological age with an estimated “biological” age; a treatment that lowers estimated biological age relative to chronological age is taken as a favourable signal, and such clocks are increasingly used as surrogate endpoints in longevity research [22]. Two lines of evidence have been advanced for semaglutide. First, large proteomic analyses of treated participants showed changes in circulating proteins linked to body-weight regulation, lipid metabolism, inflammation, and cardiovascular disease risk, with several changes persisting after adjustment for weight loss and glycaemic improvement [21]. Second, and more directly, a randomised controlled trial reported that semaglutide slowed biological ageing across multiple DNA-methylation clocks relative to placebo, with the effect persisting after adjustment for body mass index and inflammatory markers. That trial, however, was conducted in a specific population (adults with human immunodeficiency virus (HIV)-associated lipohypertrophy) and reported a preliminary, exploratory analysis [23]. Taken together, these findings represent an interesting signal rather than proof of slowed ageing.

It must, however, be interpreted with care. First, these analyses are exploratory; ageing clocks are themselves research tools whose clinical validity and responsiveness to short-term intervention are still being established [22]. Second, a change in a biomarker-derived age estimate does not prove that the rate of ageing has been altered; it may reflect reversal of disease-related or obesity-related perturbations that the clock happens to capture. Third, mortality associations from observational or post hoc analyses are vulnerable to confounding. Notably, the reported analyses themselves do not establish that the drug slows the ageing process [23]. The responsible reading is that AOMs produce favourable shifts in some ageing-associated biomarkers and reduce mortality in selected high-risk populations, but that a claim of true ageing deceleration is not yet supported.

Direct mortality evidence is nonetheless accumulating: meta-analyses and large trial programmes report reductions in all-cause and cardiovascular mortality with GLP-1 RAs in populations with cardiovascular or renal disease [4,18]. Whether these benefits extend to metabolically healthy individuals taking AOMs for longevity or appearance—a rapidly growing off-label use—is entirely untested, and the risk–benefit balance in that population cannot be assumed to be favourable.

## 6. Mechanisms Linking AOMs to the Hallmarks of Ageing

Several mechanisms have been proposed to connect AOMs to recognised hallmarks of ageing, providing the biological scaffolding for the longevity hypothesis [17]. Chronic low-grade inflammation (“inflammaging”) is attenuated by GLP-1 RAs through reductions in pro-inflammatory cytokines and improved adipose-tissue immune profiles [16,17]. Oxidative stress and mitochondrial dysfunction may be mitigated via improved metabolic substrate handling. Cellular senescence and the senescence-associated secretory phenotype have been modulated in preclinical models, and autophagy—a key proteostasis mechanism—may be enhanced. Endothelial function and vascular ageing improve, consistent with the cardiovascular outcome data. Caloric restriction itself, which AOMs may partly mimic through appetite suppression, is among the most robustly established interventions to extend lifespan and healthspan across species [24].

This mechanistic case is coherent and partly supported, but it carries an important caveat that recurs throughout this review: most of these effects could be secondary to weight loss, reduced caloric intake, and improved glycaemia rather than evidence of a distinct “anti-ageing” action. Disentangling direct receptor-mediated geroprotection from the well-known benefits of losing excess weight is the central unanswered scientific question, and it can only be resolved by trials that control for weight change.

## 7. “Ozempic Face”, “Ozempic Body”, and the Aesthetics of Rapid Weight Loss

Several terms coined by patients and amplified in the media—most prominently “Ozempic face” and “Ozempic body”—are increasingly raised during aesthetic and general consultations. None of them are a formal clinical diagnosis, and high-quality data are limited; but each maps onto a biologically predictable consequence of rapid, large-magnitude weight loss, and clinicians must be prepared to address them candidly and accurately [8,9].

“Ozempic face” describes the gaunt, hollowed, or prematurely aged facial appearance some patients develop after rapid weight loss. It is not specific to semaglutide—any rapid weight loss can produce it—but the scale and visibility of the AOM-treated population have brought it to prominence. Facial fat compartments (buccal, malar, periorbital, and temporal) provide structural support; with weight loss of roughly 15–22% of body weight, as seen in STEP and SURMOUNT [2,3], these compartments deflate faster than overlying skin can contract, producing laxity, deepened nasolabial folds, jowling, and temporal and cheek hollowing, particularly in older patients whose skin has reduced elastic recoil. The paradox is notable: a treatment marketed adjacent to “longevity” can make the face look older.

“Ozempic body” and related concerns extend the same physics to the trunk and limbs: redundant skin, breast and buttock deflation, and visible loss of muscle definition after substantial and rapid fat (and partly lean) loss. The cosmetic consequences have driven measurable demand for facial volume restoration (dermal fillers, fat grafting, and deoxycholic acid), skin-tightening procedures (energy-based devices, surgical excision after major loss), and body contouring [9]. For the aesthetic clinician, the practical message is that AOM-associated appearance change is predictable, often distressing, and frequently amenable to intervention, and that it should be discussed proactively, framed as a consequence of successful weight loss rather than a complication, and managed with realistic expectations.

A common patient question concerns reversibility. Soft-tissue volume can be partially restored, and skin laxity addressed, but the spontaneous recovery of facial volume is limited, especially in older patients and after very rapid loss. Slower, more gradual weight reduction, adequate protein intake, and resistance exercise may attenuate the severity of these changes—a point that links the aesthetic discussion directly to the lean-mass concerns considered next.

## 8. Body Composition and Cardiometabolic Remodelling

Weight, the headline number in obesity trials, is a poor proxy for the changes most relevant to ageing and appearance. What matters for healthspan is the composition of the weight lost—specifically, the preferential loss of metabolically harmful visceral and ectopic (notably hepatic) fat relative to subcutaneous fat and lean mass, and the downstream cardiometabolic remodelling that follows. A distinguishing feature of the newer multi-receptor agents, and a recurring theme at recent scientific meetings, is that they appear to act with increasing “metabolic specificity”, targeting these harmful depots rather than simply reducing the number on the scale [25,26].

The glucagon/GLP-1 dual agonists, such as survodutide, illustrate the point. In the phase 3 SYNCHRONIZE-1 trial (adults with obesity without diabetes) and the companion SYNCHRONIZE-MASLD trial (obesity with steatotic liver disease)—published together in major journals and presented at the 2026 American Diabetes Association meeting—most of the weight lost was derived from fat, with reductions of roughly one-third in visceral adipose tissue and around 60% in liver fat, alongside high rates of liver-fat normalisation; these depot-specific effects, attributed mechanistically to hepatic glucagon-receptor activation increasing fat oxidation, plausibly exceed what the moderate (~16–17%) overall weight loss alone would predict [25,26]. Earlier phase 2 work had already shown survodutide produced biopsy-confirmed improvement in metabolic dysfunction-associated steatohepatitis (MASH) without worsening fibrosis [20]. Because hepatic steatosis, visceral adiposity, and the low-grade inflammation they are central to cardiometabolic ageing, a shift in body composition of this kind is arguably more relevant to healthspan than weight loss per se.

The triple agonist retatrutide shows a similar, perhaps more pronounced, pattern. In a dedicated phase 2 MASLD substudy, liver fat fell by up to ~82% with near-normalisation in most participants, and visceral adipose tissue fell by up to ~48% at 48 weeks [27]. A separate dual-energy X-Ray absorptiometry (DXA) body-composition substudy in type 2 diabetes found that fat mass accounted for roughly two-thirds of the weight lost (fat-loss index ~64.6%), a fat-to-lean loss ratio comparable to that seen with other agents despite retatrutide’s larger absolute weight loss [28]. This is an important nuance: the greater potency of triple agonism does not, in these data, translate into a disproportionate loss of lean mass, which tempers, though does not eliminate, the sarcopenia concern discussed in the next section.

Taken together with the cardiovascular and renal outcome data reviewed earlier, these body-composition findings suggest that the cardiometabolic benefit of AOMs may be mediated in part through favourable redistribution and reduction in harmful fat depots, and that future agents are being explicitly optimised for this quality of weight loss rather than its quantity. The relevance to longevity is direct, since visceral and hepatic fat, insulin resistance, and chronic inflammation are among the principal modifiable drivers of age-related cardiometabolic disease; the relevance to aesthetics is that the depot-selective action of these agents may, in principle, shape the pattern of soft-tissue change, although this has not been formally studied.

### Targeting Harmful Fat Depots and the Facial-Fat “White Space”

A notable implication of these body-composition data is that the therapeutic value of an agent may lie less in the magnitude of weight removed than in where the fat is lost. Collectively, these findings point toward an era of more targeted weight-management therapies, agents valued for preferentially reducing visceral and hepatic fat while relatively preserving lean mass. This distinction is clinically meaningful because visceral and ectopic fat, rather than total body weight, drive much of the cardiometabolic risk associated with obesity, and because a large proportion of people with obesity also have underlying steatotic liver disease, for whom depot-selective action is the more relevant therapeutic goal than the largest possible reduction on the scale.

This same favourable body-composition profile has begun to be extrapolated, speculatively, toward an aesthetic argument: that agents producing moderate, depot-targeted fat loss might also better preserve facial fat, and so avoid the gaunt, prematurely “aged” facial appearance associated with rapid or extensive weight loss on existing therapies (Section 7). If borne out, this would address what is not only an aesthetic concern but a documented adherence barrier, since visible facial ageing contributes to treatment dissatisfaction and discontinuation. It is essential to be clear that this advantage is at present entirely hypothetical: no controlled data demonstrate that any agent preferentially spares facial fat, and the depot-selectivity shown for visceral and hepatic compartments cannot simply be assumed to extend to the face.

Facial fat preservation is, in this sense, a clear research white space—no randomised study currently assesses facial volume loss as a formal endpoint, despite its prominence in patient-facing discussion. Generating such data carries a genuine tension: formal aesthetic endpoints risk shifting the narrative toward appearance over metabolic health, an emphasis that could distort both prescribing and the framing of these drugs. Yet the same data could equally support more patient-centric communication for individuals deterred by the visible ageing effects of weight loss. The broader, and still unproven, hypothesis is that certain incretin-based therapies might enable moderate, targeted reduction of “unhealthy” fat depots while relatively sparing compartments such as facial fat—decoupling metabolic benefit from the perception of an “older” appearance. Until prospective imaging-based studies test this directly, it should be presented to patients as a plausible but unestablished possibility rather than a property of any current agent.

## 9. Lean Mass, Sarcopenia, and Healthspan

A recurring tension in the longevity framing of AOMs is that a variable fraction of the weight lost is lean (fat-free) mass, reported across trials in a wide range, from approximately 15% or less to 40–60% of total weight lost depending on the agent, the magnitude and rate of loss, and the body-composition method used [29]. This matters acutely for ageing: skeletal muscle mass and strength are strong predictors of physical function, falls, frailty, and mortality, and sarcopenia is a defining feature of unhealthy ageing. An intervention promoted for healthspan that simultaneously reduces muscle mass in older adults presents a genuine paradox that the longevity narrative tends to understate. The concern is real but should not be overstated: as noted above, the retatrutide DXA substudy found the proportion of lean-mass loss comparable to that of established agents despite greater total weight loss [28], and relative (per-kilogram) muscle strength may be preserved or even improve as fat is shed [29]. The clinically meaningful question is therefore less whether lean mass falls in absolute terms—it usually does—than whether function and relative muscle quality are maintained, particularly in older adults.

Mitigation strategies are therefore central rather than peripheral. Adequate protein intake, structured resistance training, slower dose escalation, and avoidance of excessive weight loss in already-lean older patients are all reasonable, and a pharmacological pipeline aimed specifically at preserving lean mass during weight loss (including myostatin/activin pathway agents and amylin-based combinations under study for favourable body-composition effects) is emerging [15,30]. The net effect of an AOM on healthspan in an older adult depends heavily on whether lean mass and function are protected, which argues against indiscriminate use for appearance or “longevity” in populations at risk of sarcopenia.

## 10. Skin, Wound Healing, and Dermal Biology

Beyond the volumetric effects of weight loss, AOMs may have more direct relevance to skin and dermal ageing. GLP-1 receptors are expressed in skin and dermal fibroblasts, and preclinical work suggests GLP-1 signalling can modulate fibroblast activity, collagen metabolism, inflammation, and wound healing [16]. Improved glycaemic control and reduced systemic inflammation plausibly benefit skin quality and healing in patients with obesity and diabetes, in whom impaired wound healing is well documented. Conversely, rapid loss of subcutaneous fat reduces the dermal support that contributes to a youthful appearance, and any benefit to dermal biology must be weighed against the mechanical consequences of volume loss. As with other tissues, the direct dermatological effects of AOMs in humans are largely unstudied, and current claims rest on receptor expression and mechanism rather than controlled clinical data.

## 11. Off-Label Use for Longevity and Appearance: Risks and Ethics

The growing interest in AOMs as longevity and aesthetic agents has practical and ethical consequences. Use by metabolically healthy, normal-weight, or only mildly overweight individuals—for appearance, perceived “optimisation”, or anti-ageing—is growing, yet the entire favourable risk–benefit evidence base derives from populations with obesity, diabetes, or cardiovascular and renal disease [4,18]. In lower-risk individuals, the absolute benefit is uncertain or absent, while the harms—gastrointestinal effects, lean-mass loss, facial and body soft-tissue changes, gallbladder disease, the consequences of long-term (potentially lifelong) exposure, and rebound weight gain on discontinuation—still apply [2,3,5,8,9,29,31]. Enthusiasm for “longevity” applications risks outpacing both the evidence and the regulatory framework.

A related concern is the growing use of these agents by patients with eating disorders. Their potent appetite-suppressing and weight-reducing effects make them attractive to individuals with anorexia nervosa, bulimia nervosa, or binge-eating disorder, some of whom obtain them for weight control despite the potentially serious risks in this population, including electrolyte disturbance, exacerbation of restrictive or purging behaviours, and reinforcement of the pathological drive for thinness. Closely linked to this is the observation that rapid and substantial weight loss is not the only hazard: excessive weight reduction itself may become problematic, particularly when it is driven by body-image disturbance rather than by a medical indication. For such patients, the visible soft-tissue and facial changes discussed above may intensify, rather than relieve, body-image distress. Prescribers should therefore screen actively for disordered eating and body-image pathology before initiating therapy, be alert to weight loss that exceeds any clinically appropriate target, and involve mental health and eating disorder specialists where such features are present.

Clinicians should therefore distinguish clearly between evidence-based indications and aspirational off-label use, counsel realistically about the durability of effects and the need for ongoing therapy, screen for disordered eating and body-image concerns (particularly given aesthetic motivations), and avoid endorsing “anti-ageing” claims that the data do not support. Equity considerations—cost, access, and the medicalization of normal appearance and ageing—also warrant explicit attention.

## 12. Clinical Considerations for Longevity and Aesthetic Practice

Considerations are graded using a simplified GRADE-informed scheme: (A) supported by randomised controlled trial evidence; (B) supported by observational/cohort evidence or expert consensus with mechanistic support; and (C) expert opinion or extrapolation from analogous conditions. Given the immaturity of the longevity and aesthetic evidence base, most considerations are Grade B or C, and are labelled as such explicitly rather than overstating certainty. The considerations detailed below are summarised in Table 2.

### 12.1. Indication and Patient Selection

Reserve AOMs for evidence-based indications (obesity, T2DM, and the cardiovascular/renal populations studied in outcome trials); regard use purely for longevity or appearance in metabolically healthy individuals as unproven and weigh harms accordingly [Grade C].

### 12.2. Counselling on the “Longevity” Claim

Communicate honestly that AOMs reduce cardiovascular, renal, and mortality risk in high-risk groups and shift some ageing-associated biomarkers favourably, but that true slowing of biological ageing is not established [Grade B].

### 12.3. Preserving Lean Mass

Counsel adequate protein intake and structured resistance training, escalate dose gradually, and avoid excessive loss in lean or sarcopenia-prone older adults [Grade B]:

- Regular resistance training to attenuate muscle loss [29].
- Protein intake targeting the higher end of recommended ranges during active weight loss [29].
- Monitoring of body composition (not weight alone) where feasible.

### 12.4. Aesthetic Management

Discuss “Ozempic face/body” proactively as a predictable consequence of rapid weight loss; favour gradual loss where appropriate; refer to dermatology or plastic surgery for volume restoration, skin tightening, or contouring in distressed patients [Grade C].

### 12.5. Screening and Safety

Screen for disordered eating and body-image concerns, particularly with aesthetic motivations; monitor for known adverse effects; review the plan for long-term therapy and the likelihood of rebound on discontinuation [Grade B].

### 12.6. Interdisciplinary Communication

Coordinate among prescriber, aesthetic/dermatology, nutrition, and exercise professionals so that metabolic, longevity, and appearance goals are managed coherently rather than in isolation [Grade C].

## 13. Limitations of the Current Evidence

**Figure 1:**
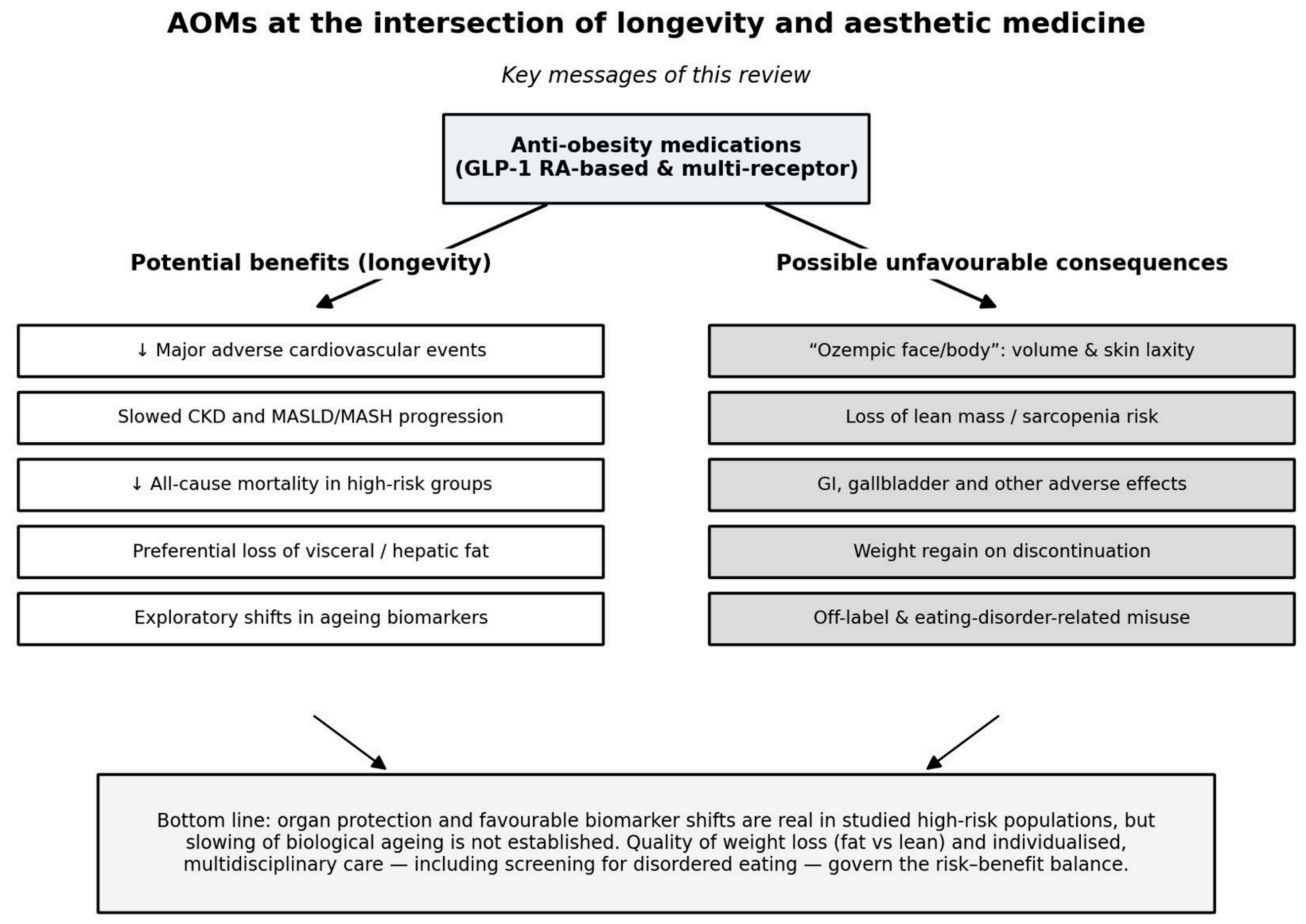
Anti-obesity medications at the intersection of longevity and aesthetic medicine: summary of the review’s key messages; ↓, reduction.

Several limitations constrain every conclusion in this review and must be stated plainly. First, dedicated human trials with longevity endpoints are essentially absent; most clinical inference is indirect, drawn from cardiovascular, renal, and metabolic trials not designed to assess ageing. Second, biological-age signals rest substantially on exploratory analyses using ageing-clock tools of still-uncertain clinical validity, and a biomarker shift does not establish slowed ageing. Third, the central confounder—separating direct receptor-mediated geroprotection from the benefits of weight loss, caloric restriction, and improved glycaemia—is rarely resolved. Fourth, the aesthetic phenomena (“Ozempic face/body”) are supported largely by clinical commentary and media reports rather than peer-reviewed prospective data, and have been treated here as hypothesis-generating. Fifth, almost no data exist for the rapidly growing off-label use in metabolically healthy, normal-weight individuals, in whom the risk–benefit balance may differ entirely. Sixth, this is a narrative, not systematic, review, and is subject to selection bias. Finally, the multi-receptor pipeline is evolving rapidly; trial figures cited here reflect data available to mid-2026 and will be superseded. Readers should weigh conclusions accordingly: the direction of evidence is encouraging, but its strength—especially for the longevity claim—is modest. The principal messages of this review, weighing the potential longevity-related benefits of these agents against their possible unfavourable consequences, including aesthetic and other treatment-related adverse outcomes, are summarised in Figure 1.

## 14. Conclusions

Anti-obesity medication—from the established GLP-1 mono-agonists and the dual agonist tirzepatide to an emerging frontier of triple agonists, glucagon co-agonists, amylin combinations, and GIP-antagonist hybrids—has reshaped metabolic medicine, and growing interest now extends to its possible roles in longevity and aesthetic medicine [2,3,4,10]. The questions this raises are genuinely interesting: these agents reduce cardiovascular, renal, and mortality risk, improve multiple organs implicated in ageing, preferentially strip the visceral and hepatic fat particularly relevant to cardiometabolic ageing, shift some ageing-associated biomarkers favourably, and visibly transform appearance.

At the same time, the human data linking AOMs directly to slowed biological ageing remain immature; most of the supporting evidence is indirect, mechanistic, or anecdotal. It is precisely this combination—strong signals across metabolism, geroscience, and aesthetics set against a thin base of direct evidence—that makes a coherent and sceptical synthesis timely, and that argues for incorporating validated ageing and body-composition endpoints into future AOM trials.

The responsible conclusion is a measured one. The mechanistic and outcome evidence for organ protection is strong in high-risk populations; the evidence that AOMs slow biological ageing itself, or that they benefit metabolically healthy people taking them for longevity or appearance, remains limited and largely indirect. The aesthetic consequences of rapid weight loss—“Ozempic face” and “Ozempic body”, and the loss of lean mass—are real, predictable, and partly manageable, and sit in tension with the youthful, healthspan-extending image the longevity framing projects. Clinicians should engage actively: selecting patients by evidence rather than aspiration, counselling honestly about what is and is not established, protecting lean mass, managing appearance change proactively, and coordinating multidisciplinary care, while neither overstating benefit nor dismissing the substantial signals that have a genuine biological basis. The field’s most pressing need is straightforward: trials that place ageing, function, and body composition—including facial volume, currently a conspicuous white space—among their endpoints, and that control for weight loss. Until then, careful clinical attention and honest communication remain the most appropriate standard of care.

## Figures and Tables

**Table 1:** Anti-obesity agents with at least preclinical evidence bearing on longevity or aesthetic/body-composition outcomes, with the specific finding, its evidence level, and the principal caveat for each. Agents whose only documented effect is weight reduction are excluded (see note).

Agent (Receptor Target)	Status	Longevity-/Aesthetics-Relevant Finding	Evidence Level	Key Caveat
Semaglutide (GLP-1)	Approved	MACE −20% (SELECT); slowed CKD progression and reduced CV/renal death (FLOW); reduced all-cause mortality; large proteomic analyses show changes partly independent of weight loss, and an exploratory randomised controlled trial (RCT) reported slowed epigenetic ageing	RCT (CV, renal); exploratory (proteomic, epigenetic clock)	Biomarker analyses exploratory; do not prove slowed ageing
Tirzepatide (GLP-1/GIP)	Approved	Non-inferior MACE-3 vs. dulaglutide with ~8% lower rate and ~16% lower all-cause mortality, plus greater renal preservation (SURPASS-CVOT, NEJM 2025); broad organ-protective profile	RCT (CV, mortality, renal)	Active-comparator (not placebo) trial; largest weight loss also drives greatest soft-tissue/lean-mass change
Liraglutide (GLP-1)	Approved	Established CV benefit (LEADER); preclinical anti-inflammatory and osteogenic/anti-senescence signalling relevant to ageing biology	RCT (CV); preclinical (mechanism)	Ageing-specific effects preclinical; superseded clinically by weekly agents
Retatrutide (GLP-1/GIP/glucagon)	Phase 3 (TRIUMPH)	Highest weight loss to date (~24%, Ph2); liver fat ↓ up to ~82% and visceral fat ↓ up to ~48% (MASLD substudy); DXA substudy shows fat-loss index ~64.6% (lean-loss proportion similar to other agents)	RCT Ph2 (weight, hepatic, DXA body composition)	Greatest absolute lean-mass loss despite comparable proportion; longevity endpoints untested
Survodutide (GLP-1/glucagon)	Phase 3	Visceral fat ↓ ~34%, liver fat ↓ ~63% with high liver-fat normalisation (SYNCHRONIZE-1/MASLD); biopsy-confirmed MASH improvement without worsening fibrosis (Ph2)	RCT Ph3 (body composition, hepatic); RCT Ph2 (histology)	Depot-specific effect partly exceeds overall weight loss; no direct ageing or aesthetic endpoints
Cagrilintide/amylin analogues (amylin–calcitonin)	Phase 2/3	Preclinical evidence of fat-mass loss with relative lean-mass preservation—directly relevant to sarcopenia/healthspan	Preclinical (body composition)	Human lean-mass-preservation data not yet confirmed
CagriSema (GLP-1 + amylin)	Phase 3 (REDEFINE)	~20.4–22.7% weight loss; amylin component hypothesised to favour body composition during loss	RCT Ph3 (weight); preclinical (composition)	Body-composition advantage in humans not yet demonstrated

Note: This Table is restricted to agents with at least preclinical evidence bearing on longevity or aesthetic/body-composition outcomes; agents whose only documented effect is weight reduction (e.g., the GIP-antagonist bispecific maridebart cafraglutide, the unimolecular GLP-1/amylin agent amycretin, and earlier-stage GIP-monotherapy are discussed in the text but deliberately excluded here to avoid implying evidence that does not yet exist). Status and findings reflect data available to mid-2026 and are subject to change. Abbreviations: CKD, chronic kidney disease; CV, cardiovascular; DXA, dual-energy X-Ray absorptiometry; MACE, major adverse cardiovascular events; MASLD, metabolic dysfunction-associated steatotic liver disease; MASH, metabolic dysfunction-associated steatohepatitis; RCT, randomised controlled trial; ↓, reduction from baseline.

**Table 2:** Summary of clinical considerations for clinicians managing patients on anti-obesity medications for longevity and aesthetic goals, with GRADE-informed strength of evidence (A, randomised controlled trial evidence; B, observational/consensus with mechanistic support; C, expert opinion or extrapolation).

Domain	Key Action	Grade
Patient selection	Reserve for evidence-based indications; treat longevity/appearance-only use in healthy individuals as unproven	C
Longevity counselling	Convey CV/renal/mortality benefit in high-risk groups; do not claim proven ageing deceleration	B
Lean mass	Protein intake + resistance training; gradual escalation; avoid excess loss in sarcopenia-prone elders	B
Aesthetics	Discuss “Ozempic face/body” proactively; gradual loss; refer for volume/skin/contouring as needed	C
Screening/safety	Screen for disordered eating/body image; monitor adverse effects; plan for long-term therapy and rebound	B
Communication	Coordinate prescriber, aesthetics, nutrition, and exercise care	C

## Data Availability

No new data were created or analyzed in this study. Data sharing is not applicable to this article.

## Abbreviations

The following abbreviations are used in this manuscript: AOM, anti-obesity medication; GLP-1, glucagon-like peptide-1; GLP-1 RA, glucagon-like peptide-1 receptor agonist; GIP, glucose-dependent insulinotropic polypeptide; GIPR, GIP receptor; T2DM, type 2 diabetes mellitus; CV, cardiovascular; MASLD, metabolic dysfunction-associated steatotic liver disease; DPP-4, dipeptidyl peptidase-4; SASP, senescence-associated secretory phenotype; GRADE, Grading of Recommendations, Assessment, Development and Evaluations.
